# Clinical Society of Maryland

**Published:** 1894-02

**Authors:** H. O. Reik

**Affiliations:** Secretary; No. 525 N. Howard Street


Secretary.

---

### CLINICAL SOCIETY OF MARYLAND.

### STATED MEETING HELD JANUARY 5TH, 1894.

The 288th regular meeting was called to order by the President, DR. J. EDWIN MICHAEL.

DR. JULIUS FRIEDENWALD gave a demonstration of the electrical illumination of the stomach. The instrument used consisted of a small incandescent electric light attached to a soft rubber tube, which enclosed the wires, and the other end of which was in connection with the battery. The stomaeh tube was first introduced and one litre of water poured into the stomach. The tube was then withdrawn and the electric light-bearing tube inserted in its stead. The room was then darkened, and when the battery was turned on the stomach was brilliantly illuminated. It was plainly outlined, the whole anterior wall was easily inspected, blood vessels of the abdominal wall being beautifully marked and the movements of the stomach during respiration well demonstrated.

DR. OSLER thought this instrument would probably render valuable assistance in diagnosing very small tumors of the anterior wall which could not easily be found by palpation.

DR. GEORGE PRESTON related an interesting case of "Widespread Muscular Atrophy."

Patient, a boy of eighteen, had been ill with cerebro-spinal meningitis for four or five weeks about four months previous to my seeing him. He was then feeling well enough and applied for treatment because of spinal curvature which caused considerable deformity and interfered with walking. The muscles of the back were wasted and the deltoid and those of the arm and thigh were gone. Reflexes, both deep and superficial, were normal. Facial muscles were not affected. There was absolute deafness. No history of pain.

Dr. Harlan mentioned a case he had seen of absolute deafness attendant upon cerebro-spinal meningitis. Patient had also had syphilis and at the time had a large polypus in one ear. The polypus was removed and potassium iodide administered. In a few days he could hear well in the other ear. It was the only case he had ever seen of absolute deafness where there was restoration to hearing.

Dr. Frank Martin reported a case of "Tubercular knee-joint cured by injection of Iodoform emulsion."

In November, 1893, I was called in consultation to see the patient, a child twenty-two months of age. Family history: Tuberculosis on mother's side.

*Child:* A picture of general tuberculosis, emaciated to a skeleton, hectic, high temperature, rapid pulse, persistent cough, lungs infiltrated, glands all over the body enlarged and knee on left side very much enlarged and full of pus.

In June the child had a cervical adenitis which was considered tuberculous. During August lobular pneumonia developed which cleared to some extent, but cough continued and was gradually followed by symptoms enumerated above. Two weeks prior to my seeing it, the knee began to swell and became painful. This steadily incread until the joint was quite filled and patella floating. It looked as if it would break on the outside. I gave doubtful prognosis, had the limb thoroughly cleansed, applied soap poultice and tied it up in bichloride. The next day operated, opened the joint freely, washed out with bichloride 1 to 2,000 and then with sol. boracic acid three per cent.; closed the wound, but before tightening the sutures injected one and a half ounces of sterilized emulsion, iodoform five per cent. I then hermetically sealed the wound, covered with sterilized dressings and put on plaster splint. Slight reaction followed. On the third day the child had only temperature of 100°.

When dressed on the tenth day the wound was healed, no fluid in the joint and joint perfectly movable. Kept up immobilization and prescribed cod liver oil and syrup iodide of iron.

To-day the joint is perfectly well, glands are decreasing in size, there is no cough and child is getting fat.

H. O. Reik, M. D.
*No. 525 N. Howard Street.* Secretary.

